# International cooperation and the challenge of internet accessibility in Caribbean territories: example of a collaborative platform between the University Hospital of Martinique and Ramón González Coro Hospital in Cuba, through the French PRPH-3 program

**DOI:** 10.1186/s12909-023-04321-1

**Published:** 2023-05-19

**Authors:** Rémi Houpert, Thierry Almont, Christian Mésenge, Line Kleinebreil, Laurence Forlini, Bruno Magnone, Vincent Leroux, Mylène Vestris, Christelle Montabord, Jaylin Carmenate, Yaima Galán, Maria Caredad Rubio, Rodolfo Enriquez, Carol Burte, Nicolas Gatimel, Louis Bujan, Norelyakin Kara, Olivier Edwige, Éric Huyghe, Clarisse Joachim, Jacqueline Véronique-Baudin

**Affiliations:** 1 Research and Development in Oncology Unit UF3596, Cancerology Department, Martinique University Hospital (CHU Martinique), Fort-de-France, Martinique; 2 Cancerology Department, General Cancer Registry of Martinique UF1441, Martinique University Hospital (CHU Martinique), Fort-de-France, Martinique; 3 Oncosexology Unit, Cancerology Department, Martinique University Hospital (CHU Martinique), Fort-de-France, Martinique; 4 World Francophone Digital University, UNFM / HNSM 14 rue du Val d’Osne, Saint-Maurice, 94450 France; 5 Hospital Ginecobstétrico Ramón González Coro, La Havane, Cuba; 6 National Cancer Registry of Cuba, National Institute of Oncology and Radiobiology, La Havane, Cuba; 7 French Society of Sexual Medicine (SFMS), Lille, France; 8 Embryonic Development, Fertility and Environment Laboratory (DEFE) UMR 1203, Toulouse 3 Paul Sabatier University, Toulouse, France; 9 Urology Unit, Cancerology Department, Martinique University Hospital (CHU Martinique), Fort-de-France, Martinique; 10 EDEO Technologies, 11 rue des Arts et métiers, Fort-de-France, 97200 Martinique; 11 French Education and Research Group in Andrology, Urology and Sexology (GEFRAUS), Reproductive Medicine Department, Paule de Viguier University Hospital, 330 avenue de Grande-Bretagne, Toulouse cedex 9, 70034, 31059 TSA France; 12 Francophone Association for Supportive Care (AFSOS), 76, rue Marcel Sembat, Bègles, 33130 France

**Keywords:** Low bandwidth, Digitalization, Innovation, e-learning, Caribbean cooperation

## Abstract

**Background:**

Martinique shares with the other Caribbean countries specific public health issues, particularly in the diagnostic and therapeutic management of cancer patients. Mutualization of human and material resources by promoting cooperation is the most appropriate response to the challenges of the health systems of the Caribbean territories. Through the French PRPH-3 program, we propose to set up a collaborative digital platform adapted to the specificities of the Caribbean to strengthen professional links and skills in oncofertility and oncosexology and reduce inequalities in access to reproductive and sexual health care for cancer patients.

**Methods:**

Within the context of this program, we have developed of an open-source platform based on a Learning Content Management System (LCMS), with an operating system developed by UNFM for low speed internet. LO libraries have been created and interaction between trainers and learners were done in asynchronous mode. This training management platform is based on: a TCC learning system (Training, Coaching, Communities); a web-hosting with pedagogical engineering appropriate to low bandwidth; a reporting system and a responsibility for processing.

**Results:**

We have carried out a flexible, multilingual and accessible digital learning strategy functionality called e-MCPPO according to low-speed internet ecosystem. In close connection with the e-learning strategy we conceived (i) a multidisciplinary team; (ii) an appropriate training program for expert health professionals and (iii) a responsive design.

**Discussion and Conclusion:**

This low-speed web-based infrastructure allows communities of experts to cooperate in creating, validating, publishing and managing academic learning content. The self-learning modules provide the digital layer for each learner to extend their skills. Learners, as well as trainers, would gradually take ownership of this platform and encourage its promotion. Innovation in this context is both technological (low-speed Internet broadcasting, free interactive software) and organizational (moderating educational resources). This collaborative digital platform is unique in its form and content. This challenge could contribute to the digital transformation of the Caribbean ecosystem for capacity building in this specifics topics.

## Context

Martinique shares specific public health problems with other Caribbean countries, particularly in cancer patients’ diagnostic and therapeutic management. Therefore, the University Hospital of Martinique (CHUM) has been involved in medical cooperation in oncology within the Caribbean to improve skills and knowledge in this field and to promote the creation of bilateral relations to improve the management of patients with cancer [1, 2] Furthermore, because of their membership in Caribbean health organizations such as PAHO, CARPHA, and OECS, the Oncology Hematology Urology Department teams wanted to promote and share this French expertise in the Caribbean. Indeed, cooperation in public health and oncology is currently a significant issue for the island of Martinique, given its geopolitical position in the Caribbean region. [3]

In order to support cooperation initiatives, France has set up a “Hospital Network and Partnership Projects” (PRPH) program to finance collaborative projects between a French hospital and a foreign hospital identified. This program contributes to improving the quality of care and management of developing hospitals. This joint program of the French Development Agency (AFD) and the French Hospital Federation (FHF) is based on the observation that hospitals play a significant role in the health systems of developing countries, both as providers of care and as places for training health professionals. The current project is being carried out within the framework of PRPH 3 to improve the quality of care and the development of oncofertility and oncosexuality management at the Ramón González Coro Hospital in Cuba with the Pierre Zobda-Quitman University Hospital in Martinique as the French counterpart. [4]

Sexuality and fertility disorders are frequent and long-lasting sequelae of cancer and its treatment.[5] Therefore, it is recommended that French institutions accredited for oncology include in the personalized care of the patients concerned the possibility of accessing oncosexuality oncofertility support care [6] following national recommendations of good practice. [7, 8] These national guidelines apply throughout the French territory, including the French departments of America that are Guadeloupe, Martinique, and Guyana. As such, at the CHUM, this care has been organized and provided since April 1, 2019, via a dedicated Unit for the oncosexuality care pathway, and in partnership with the Caribbean Center for Reproductive Medicine of the University Hospital of Guadeloupe and the local private center for medically assisted procreation, for the oncofertility care pathway.

A digital collaborative platform seemed to be the most appropriate solution for the Caribbean ecosystem due to the specificities of the islands. Nevertheless, the implementation of such a platform implied taking into account the difference in Internet penetration rates (77% vs. 40%) and broadband accessibility (71% vs. 5%) between the two islands. [4]

This cooperation program is based on a solid experiential base of identified partners. The expertise of the UNFM in accessing digital resources in “low-speed” Internet was necessary for the implementation of the project. The UNFM was initially a program developed on the African Virtual University (AVU) model and launched in 2003 by the Foundation for Policy Innovation (Fondapol). Since 2005, the UNFM has become an independent, non-profit digital NGO whose mission is to use new technologies to help fight against inequalities in access to knowledge and support professionals working in areas of scarcity or conflict. In addition, the official French experience in oncofertility and oncosexuality, respectively more than 45 years and 20 years, is also a significant asset.

In this context, all the experts involved in this program reviewed the available advanced technologies for multidimensional training generation and integration in an open-living lab science approach as well as clinical and practical application. This network system would be the mains issue and opportunity for the future of Caribbean ecosystem. [9, 10]

Bringing local expert health services closer together through digital technology will help to reduce geographical disparities by guaranteeing simple and equitable access and delivery of care.

The challenges have been clearly identified for this project:

- Promote digital technology to modernize and improve the efficiency of the healthcare system, even in times of health crisis;
- Support the co-construction of digital solutions with partners identified in accordance with their ecosystems;
- Facilitate synergies between digital structures in order to build a multi-center territorial hospital community characterized by a shared history and culture, where healthcare systems are no longer juxtaposed, but are organized into expert local health territories;
- Create ad-hoc training programs for healthcare professionals around digital technology.

In order to respond to the insular states’ challenges, we propose to set up a collaborative and multilingual digital platform adapted to the specificities of the Caribbean to strengthen professional links and skills in oncofertility and oncosexology and reduce inequalities in access to reproductive and sexual health care for cancer patients. This solution guarantees a perennial, simple and free access in low-speed internet, taking into account innovative digital technologies, on any type of device for capacity-building activities in the Caribbean.

## Methods

Within the context of this program, we have set up a collaborative platform adapted to the Caribbean ecosystems focusing on oncosexology and oncofertility area.

### Fundamental principles of infrastructure used

We have implemented a multilingual open source platform based on a Learning Content Management System (LCMS) named e-MCPPO, hosted in Switzerland, with an operating system developed by the UNFM. This LCMS model is based on the LO (Learning Objects) model composed of learning objectives, assessments and content. LO libraries have been created according to the course format (PowerPoint slides, Word documents, html, pdf, …). Interaction between trainers and learners is done in asynchronous mode (audio/videoconference, dialogue) [11].

### A pedagogical engineering (supported by the UNFM) made up of 2 components

#### Course recording software with live compression

The courses are adapting to mobility (responsive design) and put online directly by the trainer thanks to the DUDAL application, which is very easy to use and interactive. The streams of the courses are multtilingual made with subtitling function of the audio, and immediately compressed and put online with a guaranteed low speed access. DUDAL allows a web hosting with educational engineering including appropriate applications for online authoring tools to produce courses, tests and multimedia content. Remote assistance and support is provided by a UNFM technician for configuration, testing and initial use of the software. This assistance guarantees technical assistance to the experts for the recording of the courses and computer processing for the posting of the courses on the website.

### A dedicated website for sharing knowledge

We have implemented an e-learning strategy based on an asynchronous training method using a Training, Coaching and Community (TCC) process. The TCC process is defined as follows: (i) Training: All opportunities will be provided to learners as well as teachers based on the principle of empowerment. (ii) Coaching: interaction between learners and trainers in asynchronous mode (video, audio, dialogues, academic exchanges) (iii) Communities: health professionals identified and affiliated with a community of practice network of experts in oncofertility and oncosexuality are involved in this website. The sharing of skills, initiatives and collaborative work practices will be privileged and encouraged on this platform.

The content of the courses is aligned with the requests formulated by the learners or the speakers. These requests are recorded and analyzed by ad hoc surveys via the Sphinx application, which is also used to generate the questionnaires.

A trainer for this platform is defined as a team of university teachers who are responsible for the courses. Faculty teams create standard and individualized learning paths that incorporate multimedia learning resources on oncofertility and oncosexuality. The learner is defined as an identified health care professional affiliated with a network of oncofertility and oncosexuality experts. The learner can visit the website, download educational content, and manage and organize their digital course time at their own pace. Each status has different rights and privileges within e-MCCPO.

Access to the platform will be carefully controlled through a secure search portal with full traceability features.

The platform will be monitored using two indicators (a) an educational resource to report on access (number of accesses, type of access, and status of those connected), and (b) a statistical resource to assess training needs and geolocation of learners.

## Results

Collaboration with partners and expert communities of practice has enabled us to use the advanced technologies available for the generation and integration of oncosexology and oncofertility data through an easily accessible and flexible platform, in an open science approach with opportunities for the future.

**Fig. 1:**
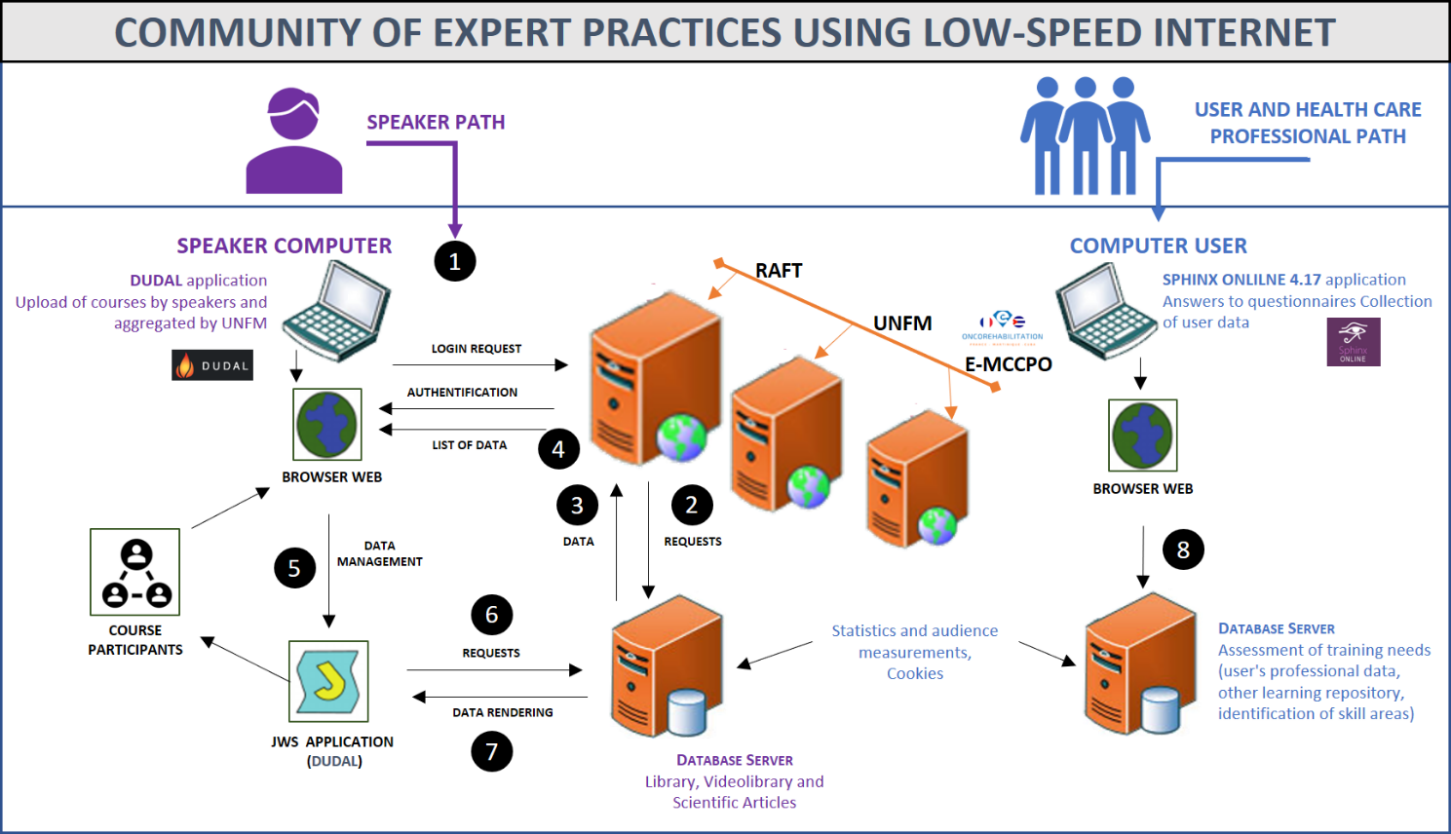
e-MCCPO Infrasctructure Diagram

**E-MCPPO: a flexible and accessible digital learning strategy (**Fig. 1**)**.

### Fundamental principles of infrastructure used

A completely secure environment with strong and easy-to-use interfaces has been created for online access. The platform content is in compliance with the European data security rules for the intellectual protection of online content (RGPD).

### Pedagogical engineering (supported by the UNFM)

The platform offers a collaborative library in the format of online courses, practical videos, tutorials, and learning resources in Oncofertily and Oncosexuality. The e-learning devices are declined in 2 main paths: a speaker path and a user/health care professional (Fig. 1).

- Digitalization to share knowledge:

Access to the LO is managed and organized by the trainer but the learner has their own digital class time. Trainers and learners communicate individually or in groups, creating discussion topics. Identified issues are managed by the platform administrator and submitted to the expert community of practice established by the program. A trainer can become a learner and a learner can become a trainer.

(b) A multidisciplinary team:

For this program, the challenge was on the one hand to identify a team with recognized experience in technology and on the other hand engineering in low bandwidth and a network of skills for sharing experiences and cooperation in sexual and reproductive health.

(c) An appropriate training program for expert health professionals:

The pedagogical contents are elaborated with the help of the feedback from the survey carried out by an online questionnaire that identified the specific know-how and needs of the learners. This training is primarily intended for all expert health professionals and anyone who, in the course of their duties, will need to enrich their knowledge in the field of sexual and fertility rehabilitation.

Remote assistance and support for software configuration, testing, tutoring sessions, and quiz statistics will be provided by the UNFM technical team.

The program is permanently accessible to the registered persons and is broadcasted at low speed from a periodically enriched platform and accessible directly online or offline mode.

The interactive aspect of the platform is ensured by active gamification in learning in the form of quizzes before and after each course session, for example.

This training will allow the learner to progress at his own pace and be sanctioned by a certificate or a diploma delivered by the French hospital.

- A responsive design:

Through innovative tools, the digitization of training will allow the provision of courses in the form of modules. The educational resources will be adapted for quality access, even at reduced Internet speeds. They will be able to benefit from a system that will synchronize the recording, the multilingual subtitling of the audio streams, and the live compression of the courses with specific software, DUDAL, from any Internet connection. DUDAL is the ideal tool used to put the courses online. The video conferences are produced with this free software developed for RAFT (Network in Francophone Africa for Telemedicine) to work with low bandwidth connections (25 kb/s) [12]. It allows participating in educational sessions either as a listener or speaker, with the possibility of an interactive forum. With quick and easy training, the system allows experts to record their presentations without traveling, with easily accessible hardware (computer, webcam and microphone) and even a weak internet connection. Technically, DUDAL converts an OpenOffice or PowerPoint presentation into HTML documents, and has the ability to run on a low bandwidth network and reconnect easily in case of interruption [13, 14].

1. A login request by username/password is asked. The application server authenticates the user.
2. The application server interacts with the database server via queries (SQL or file exchange).
3. The database server responds by sending the result of the queries as data.
4. A list of data is then sent to the speaker’s web browser.
5. The speaker can upload the courses via a link proposing a file in JNLP (Java Network Launching Protocol) format, this protocol allows the execution of a Java applet thanks to the JWS services.
6. The Java application interacts with the database server.
7. The database server transfers to the applet the rendering of the data to be proposed to the end users.
8. Access to the Sphinx Online software storage of related data such as statistics, sessions….
9. Participants access the courses through their web browser by clicking on a JNLP link.

## Discussion

This project promotes peer-to-peer learning by allowing learners to learn on their terms and progress at their own pace. Learners, like trainers, are engaged in the training process (empowerment). This strategy, to quickly answer a relevant question, will open the door to new training opportunities: (i) enabling the declaration of training needs, (ii) sharing knowledge, and (iii) expertise to create content online, quickly, easily, inexpensively with widespread access to low-speed internet. All growth opportunities will be implemented with the co-construction of digital solutions with identified partners and perfect coherence with ecosystems and low-speed internet configurations. Within the framework of e-MCCPO, teams identified in partnership with the UNFM will be central to a community of practice expert that contributes to driving change in the Pan American area. Relevant indicators will be generated by the general cancer registries of both countries involved for the first time in this atypical field.

Digital technologies will bring about significant changes in the organization and functioning of our healthcare systems. They will allow us to modernize current organizations and consider radically new and more efficient practices, especially if a new health crisis such as the COVID-19 crisis occurs again. In the Caribbean basin, bandwidth connection speeds may be limited. Nevertheless, providing solutions that maintain the responsiveness of a platform for access to training resources are innovative alternatives for these regions. Thanks to the experience of the Université Numérique Francophone Mondiale (UNFM) in providing e-learning resources accessible at very low Internet speeds, the learning platform we propose has a solid experiential base for providing quality education online in an environment of shortage. [15]

Insofar as digital technology unleashes an immense potential for “proximity” innovation, healthcare professionals (innovators) will be able to adapt their clinical practice, modernize their communication or training methods, and exchange or obtain expert advice on complex clinical cases.

In the last few years, many innovative learning programs using RAFT have shown great success in the accessibility and diffusion of courses in areas of shortage [16]. The results of Dr. GUINDO Fatoumata SISSOKO’s thesis, which evaluated the Malian experience on medical distance learning broadcasted by RAFT, have confirmed these observations. DUDAL is then the solution to support the digital learning strategy of this project in order to offer a low-cost and accessible educational option. [17] Regarding e-learning for medical education in low-resource countries, our digital strategy includes the suggestions in Sandra Barteit’s article [18]. As well, we propose (i) a thematic database on oncofertility and oncosexuality, (ii) a standardized and widely used framework for evaluating e-learning programs using the UNFM, and (iii) structured programs that integrate e-learning between recognized scientific institutions (CECOS, GEFRAUS, Toulouse University).

New horizons will open up for our scientists and physicians regarding training and learning aligned with an innovative digital strategy. These innovations are necessary to overcome the difficulties of coordination and training between local professionals. Related work will contribute to a continuum for implementing an ad-hoc training library. In addition, the innovators’ affiliation centers will be identified in the Caribbean as centers of competence and excellence in sexual and reproductive health care with high standards of clinical practice. This program can be replicated in other world regions, thanks to its low-cost interactivity and UNFM’s global expertise in deploying training in developing countries. In this dynamic setting, the health care system will also have to evolve, if not revolutionize, in terms of organization and management. Content distribution and targeting are levers for optimizing site traffic and generating qualified leads.

Digitalization of audio-visual pedagogical resources combined with the performances of the networks provided by the RAFT offer many possibilities. Indexing such resources is a major challenge for expert practice community. Furthermore, the provision of high-tech equipment and the expertise of the UNFM will generate a network of community exchanges for continuing professional development and reinforcement of health behaviors as close as possible to the local health territories.

This project responds to the national priorities of Cuban and French health policies (cancer plans and national sexual health strategies). It will be implemented in liaison with the Cuban Health Agencies and learned societies and the French Embassy in Cuba. The project and its impact will be regularly evaluated by the Martinique and Cuba-INOR cancer registries. This proposal is a extension of the collaboration initiated with Cuba for the development of bilateral scientific and medical actions. Oncofertility and oncosexology care must be better integrated in the assessment of care in our regions. This is a new area of legal recommendations to be included in the standards of care.

Access to care in terms of a digital healthcare offer must be strengthened and developed in our territories even more than elsewhere. This complementary organization, part of a global and integrated digital logic, must ensure, thanks to the PRPH-3 cooperation program and complementary programs, standardized treatment paths according to institutional recommendations (INCa and MINSAP). We are currently working to contribute to a quality and functional cooperation network, fully involved, bringing expertise and skills through a care network for complex cases, workshops, e-learning, and practical training on expert sites.

Thanks to the PRPH-3, the furtherance of such collaborative projects with Cuba, will allow to identify and generate in a more precise way in the Caribbean region, clinical, demographic, socioeconomic or organizational determinants, at the origin of the heterogeneity of the medical assistance to procreation and sexual rehabilitation. The related work of this collaborative digital platform will contribute to the establishment of a continuum for cancer surveillance and associated Oncorrehabilitation, ultimately proposing a coherent value chain, federating health professionals around adapted training, expert medical management and shared know-how, for the benefit of patients and health professionals. Within this dynamic, each country will be identified as a center of competence and excellence in onco-rehabilitation.

## Conclusion

Innovation in this context is both technological (low-speed Internet broadcasting, free interactive software) and organizational (moderating educational resources). This collaborative digital platform is unique in its form and content. Through bilateral exchanges, French and Cuban professionals will share their expertise in fertility preservation and sexual rehabilitation after cancer. However, synchronous workshops with additional face-to-face training, supervised by expert professionals, would still be suitable for the development of an ongoing network of communities of expert practice.

Spearheading the digitalization of learning, this project integrates digital tools (synchronous and asynchronous) and at the same time raises awareness of good practices in relevant thematic. The dynamic of the project allows for the transfer of accessible, innovative, and atypical operational know-how to the benefit of co-development and decentralized cooperation, supervised by French institutions and their networks of expert communities of practice. Moreover, this strategy, where teams are connected through digital health and research networks, will reinforce the French leadership in these territories.

The self-learning modules provide the digital layer for each learner to extend their skills. Learners, as well as trainers, would gradually take ownership of this platform and encourage its promotion. This challenge could contribute to the digital transformation of the Caribbean ecosystem.

## Data Availability

The datasets generated during the current study are not publicly available due the General Data Protection Regulation but are available from the corresponding author on reasonable request.

## List of abbreviations

AFD: Agence Française de Développement (French Agency for Development)
AVU: African Virtual University
CARPHA: Caribbean Public Health Agency
CECOS: Centre d’Etude et de Conservation des Oeufs et du Sperme humains (Center for the study and conservation of human eggs and sperm)
CHUM: Centre Hospitalier Universitaire de la Martinique (University Hospital of Martinique)
E-MCCPO: e-Martinique Cuba Collaborative Platform in Oncorehabilitation
FHF: Fédération Hospitalière de France (French Hospital Federation)
GEFRAUS: Groupe d’Etude, de Formation et de Recherche en Andrologie, Urologie et Sexologie (Group of Study, Training and Research in Andrology, Urology and Sexology)
JNLP: Java Network Launching Protocol
LCMS: Learning Content Management System
LMS: Learning Management System
LO: Learning Objects
MINSAP: Ministerio de Salud Pública CUBA (Ministry of Public Health)
NGO: Non-Governmental Organization
RGPD: Règlement Général sur la Protection des Données (General Data Protection Regulation)
OECS: Organisation os Eastern Caribbean States
PAHO: Pan American Health Organization
PRPH: Projet Réseaux et Partenariats Hospitaliers (Hospital Networks and Partnerships Project)
RAFT: Réseau en Afrique Francophone pour la Télémédecine (Network in Francophone Africa for Telemedicine)
TCC: Training, Coaching, Communities
UNFM: Université Numérique Francophone Mondiale (World Francophone Digital University)
